# Interspecies Insertion Polymorphism Analysis Reveals Recent Activity of Transposable Elements in Extant Coelacanths

**DOI:** 10.1371/journal.pone.0114382

**Published:** 2014-12-03

**Authors:** Magali Naville, Domitille Chalopin, Jean-Nicolas Volff

**Affiliations:** Institut de Génomique Fonctionnelle de Lyon, Ecole Normale Supérieure de Lyon, Lyon, France; Laboratoire Arago, France

## Abstract

Coelacanths are lobe-finned fish represented by two extant species, *Latimeria chalumnae* in South Africa and Comoros and *L. menadoensis* in Indonesia. Due to their intermediate phylogenetic position between ray-finned fish and tetrapods in the vertebrate lineage, they are of great interest from an evolutionary point of view. In addition, extant specimens look similar to 300 million-year-old fossils; because of their apparent slowly evolving morphology, coelacanths have been often described as « living fossils ». As an underlying cause of such a morphological stasis, several authors have proposed a slow evolution of the coelacanth genome. Accordingly, sequencing of the *L. chalumnae* genome has revealed a globally low substitution rate for protein-coding regions compared to other vertebrates. However, genome and gene evolution can also be influenced by transposable elements, which form a major and dynamic part of vertebrate genomes through their ability to move, duplicate and recombine. In this work, we have searched for evidence of transposition activity in coelacanth genomes through the comparative analysis of orthologous genomic regions from both *Latimeria* species. Comparison of 5.7 Mb (0.2%) of the *L. chalumnae* genome with orthologous Bacterial Artificial Chromosome clones from *L. menadoensis* allowed the identification of 27 species-specific transposable element insertions, with a strong relative contribution of CR1 non-LTR retrotransposons. Species-specific homologous recombination between the long terminal repeats of a new coelacanth endogenous retrovirus was also detected. Our analysis suggests that transposon activity is responsible for at least 0.6% of genome divergence between both *Latimeria* species. Taken together, this study demonstrates that coelacanth genomes are not evolutionary inert: they contain recently active transposable elements, which have significantly contributed to post-speciation genome divergence in *Latimeria*.

## Introduction

Coelacanths are lobe-finned fish that have been considered extinct since the Late Cretaceous period about 70 million years (my) ago, until a first living specimen, *Latimeria chalumnae*, was discovered in 1938 in South Africa by Marjorie Courtenay-Latimer [1]. From an evolutionary point of view, coelacanths occupy like lungfishes a key phylogenetic position between ray-finned fish and tetrapods at the basis of the sarcopterygian lineage. Their fleshy fins, which resemble the limbs of land animals, make them a pertinent model to study the water-to-land transition. A second coelacanth species, *Latimeria menadoensis*, was subsequently discovered in 1997 in Indonesia, with the capture of two individuals [2]. While coelacanths formed a highly spread taxonomic group during the Devonian [3], [4], both extant species are nowadays endangered, with only few inventoried individuals (about 300 for *L. chalumnae* [5]). Despite their geographical remoteness, *L. chalumnae* and *L. menadoensis* present a high degree of nucleotide identity at the genomic level [98.7%, based on the comparison of 20 Bacterial Artificial Chromosomes (BACs) from *L. menadoensis* with their orthologous sequences in the *L. chalumnae* genome], as well as for exons (>99.7%, based on the comparison of liver and testis transcriptomes from both species) [6], [7]. The recent genome analysis performed by Nikaido et al. even proposed a genetic divergence as low as 0.18% between the nuclear genomes of both species [8]. Such an identity rate of 98.7% at the genomic level is similar to that measured between human and chimpanzee. Considering the faster evolution in the primate lineage, the divergence time between the two coelacanth species was approximated at slightly more than 6–8 million years [6].

With fossils dating back to 300 million years that look very similar to extant animals, coelacanths have been placed by some authors in the arguable class of “living fossils”, which are characterized by a long stasis in their phenotypic evolution [1], [9]. The careful analysis of paleontological data, however, has recently challenged this picture [10]. The apparent morphological stasis has often been proposed to rely on a very slow genomic evolution [10]–[12]. While several studies based on the analysis of particular gene families such as *Hox* or protocadherins already suggested a slow evolutionary rate [13]–[15], the recent availability of genomic data allowed addressing this question in a more systematic way. By analyzing 251 protein-coding genes, which form the most constrained part of the genome, Amemiya et al. showed that these sequences evolve more slowly in coelacanth than in lungfish, chicken and mammals, with a substitution rate being half of that in tetrapods [6]. This is corroborated by the slow rate of nucleotide substitution demonstrated by Nikaido et al. based on the calculation of Ka/Ks ratios between 4,531 genes of *L. chalumnae* and *L. menadoensis* [8]. Analysis of coding sequences seems thus to sustain the idea of a slowly evolving genome.

Transposable elements (TEs) constitute a major source of genome diversity and evolution. These sequences, which are generally repeated, are able to integrate into new locations in genomes. TEs are sorted in several classes, orders and families according to their structure and mode of transposition [16]. Retroelements (class I elements) retrotranspose through the reverse transcription of an RNA intermediate into a cDNA copy, which is inserted somewhere else in the genome (copy-and-paste mechanism). Transposition of class II elements (DNA transposons) does not require any reverse transcription step: these elements generally excise and reinsert into a new locus (“cut-and-paste” mechanism). Both classes are further subdivided into orders and (super)families. TEs can be either autonomous or non-autonomous: non-autonomous elements, such as class II MITEs (Miniature Inverted–repeat TEs) and class I SINEs (Short Interspersed Nuclear Elements) do not encode the enzymes necessary for their transposition, but instead use the machinery of an autonomous protein-coding element to achieve transposition.

Originally, TEs have been relegated to parasitic “junk DNA”, with occasional negative effects on host genes such as insertional disruption, rearrangement and silencing [17], [18]. More recently, converging studies have uncovered major roles of TEs in the evolution of genes, genomes and organisms [19]. TEs are driving forces of genome plasticity: their copies, interspersed along the chromosomes, can recombine and promote genomic rearrangements such as deletions, duplications, inversions and translocations [20], [21]. In addition, TEs can duplicate and shuffle host coding sequences, and provide material for new regulatory elements (promoters, enhancers and splicing sites), new exons and even new genes – an evolutionary process called molecular domestication [22]–[25].

Considering the important impact of TEs on genome evolution, a strongly reduced transposition activity has been proposed for the “living fossil” coelacanths [21]. It was recently shown that the *L. chalumnae* genome contains 25–50% of TEs, including retrotransposons (with a high proportion of non-Long Terminal Repeat [non-LTR] retrotransposons), endogenous retroviruses and DNA transposons [6], [8], [26]. Four major bursts of transposition that principally involved LINE1, LINE2, CR1 and Deu non-LTR retrotransposons were detected through copy divergence analysis in the genome of *L. chalumnae*, supporting activity of TEs in the *Latimeria* lineage [6], [26]. Analysis of RNA-seq data showed that 14 TE superfamilies are expressed in coelacanth tissues, with a high representation of the CR1 LINE and LF-SINE families [6], [27]. Although these results suggested TE activity in coelacanths, direct evidence of recent transposition was still missing.

In this study, we have looked for the presence of TE insertion polymorphisms in orthologous regions from the genomes of the two extant coelacanth species, *L. chalumnae* and *L. menadoensis*. Identification of species-specific insertions for the CR1 LINE family as well as for other types of TEs indicated recent transposition activity in the genus *Latimeria* and showed that TEs have significantly contributed to genome divergence between both coelacanth species.

## Materials and Methods

### Origin of genomic sequences

The *L. chalumnae* genome was downloaded from the Ensembl server (http://www.ensembl.org/; accession LatCha1 GCA_000225785.1). *L. menadoensis* BAC sequences were obtained from NCBI (http://www.ncbi.nlm.nih.gov/) with following accession numbers: GI:164698640, GI:170514516, GI:189459217, GI:190886531, GI:193083250, GI:237406519, GI:239735715, GI:239835822, GI:239835823, GI:239835824, GI:239835825, GI:239835826, GI:239835827, GI:239835828, GI:239835829, GI:239835830, GI:305644147, GI:305644148 [Birren 2009, NCBI direct submissions], GI:166987259 [28], GI:220898172 (*HoxA* gene cluster), GI:220898186 (*HoxB* gene cluster), GI:220898198 (*HoxC* gene cluster), GI:220898210 (*HoxD* gene cluster) [13], GI:296011776 [29], GI:407080572 (*IgW2* locus), GI:407080573 (*IgW1* locus) [Saha 2012, NCBI direct submissions], GI:40789109, GI:50253612, GI:50253613, GI:50284579, GI:50284580, GI:50284581, GI:52077680 [Grimwood 2004, NCBI direct submissions], GI:66912372 [Lau 2010, NCBI direct submission].

### Identification of TE insertions

In order to determine orthology relationships between *L. menadoensis* BAC clones and the *L. chalumnae* genome, sequence comparison was performed using MegaBlast [30]. Best hits were selected and the main alignment diagonals were used to define the maximum match coordinates along with their orientation. Orthologous fragments are listed in Table S1. *L. chalumnae* and *L. menadoensis* TEs were localized using the RepeatMasker software [31] with a TE library specifically built for the *L. chalumnae* genome [6], [26]. TEs were selected according to the following criteria: length ≥100 nucleotides (nt) and divergence to the consensus sequence from the library ≤20%. Low complexity sequences, simple repeats as well as tRNA and rRNA (pseudo)genes were discarded. Remaining elements located in corresponding *L. menadoensis* BACs and *L. chalumnae* genome scaffolds were then listed “face to face” as shown in Figure S1, and further manually aligned to visualize orthologous insertions in both species. Species-specific insertions were inspected manually by extracting and comparing both “empty” and “filled” sites using the Muscle alignment software [32].

### Counting of shared TE insertions

Using RepeatMasker, masking of *L. menadoensis* with the *L. chalumnae* TE library might lead to artifactual split insertions in *L. menadoensis* due to discrete interspecific sequence differences with *L. chalumnae* TEs. This would lead to an overestimation of the number of common insertions. To avoid this problem, the number of common insertions between both *Latimeria* species was estimated on *L. chalumnae* data. Close hits matching contiguous parts of the same TE sequence from the database and indexed by a common element number in RepeatMasker outputs were counted as a unique insertion. The same procedure was applied on the whole genome of *L. chalumnae* to estimate its global TE content.

### Annotation of species-specific TE insertions

TE insertions were assigned to known TE superfamilies based on Wicker's classification by combining comparative and predictive approaches [16]. In order to look for putative coding regions, TE sequences were submitted to the *de novo* gene prediction program Genscan [33] and to a BlastX search [34] on the NCBI website with default parameters against the Genbank non-redundant protein sequence database. Insertions were also analyzed with the Censor software [35] that compares sequences with the Repbase repeat database [36]. Structural features such as Long Terminal Repeats (LTRs) and Terminal Inverted Repeats (TIRs) were identified with the Blast bl2seq utility [34]. Target Site Duplications (TSDs), which correspond to the duplication of few nucleotides from the insertion site, were searched at the extremities of insertions. TE expression was analyzed by sequence similarity analysis of a transcriptome of *L. menadoensis* testis [6] using the Blast algorithm with default parameters. Copy number of the identified TEs was determined in compared genomic sequences by sequence similarity using the same procedure, with filters of minimal length (80% of the considered TE length) and minimal nt sequence identity (80%) classically used to define TE families [16].

### Sequence alignments and phylogenetic reconstructions

Sequences not unambiguously associated to particular TE families were classified using phylogenetic analysis. TE sequences were extracted from BACs and genomic segments and aligned using Muscle [32] with default parameters. Phylogenetic trees were constructed with PhyML [37] using Maximum Likelihood and aLRT values (non-parametric bootstraps) on different types of sequences depending on the element analyzed: reverse transcriptase (RT) and integrase core domain protein sequences for Endogenous RetroViruses (ERVs), RT protein sequences for CR1/L2 non-LTR retrotransposons, and nucleotide sequences for SINE elements. Using the same procedure, a molecular phylogeny was also reconstructed for CR1 elements with nucleotide sequences.

## Results

We searched for the presence of species-specific TE insertions in *Latimeria* by comparing 36 BAC clone sequences from the Indonesian coelacanth *L. menadoensis* with orthologous regions from the recently published genome of its African congener *L. chalumnae* [6]. *L. menadoensis* BAC clones have a median length of 170 kb and correspond to loci of particular interest including the *IgW1* genes (immunoglobulin heavy chain) [Saha 2012, NCBI direct submission] and the *Hox* genes [13]. The analyzed sequences, which cover ca. 5.7 Mb (0.2%) of *L. chalumnae* draft genome, contain 3,063 identifiable insertions of TEs common to both species (15.7% of the fraction of the genome analyzed), corresponding to a density of approximately 540 TEs/Mb. As a comparison, we estimated the average TE density across the whole genome at 800 TE/Mb. The regions analyzed here are thus slightly depleted in TEs compared to the rest of the genome.

### Identification and characterization of species-specific TE insertions

Using a comparative approach, we searched for species-specific insertions, i.e. for sites “filled” by a TE insertion in one species orthologous to “empty” sites (i.e. without insertion) in the other (Table 1, Table 2 and Table S2). Of note, for DNA transposons, what we define here as an “insertion” in one species could alternatively be the result of the excision in the other species of an element inserted in the last common ancestor of both species. Furthermore, insertion polymorphism might reflect transposition in one species after speciation, but also insertion polymorphism at allelic positions in the last common ancestor. In any case, we consider these different possibilities as evidence for relatively recent (<10 my old) transposition events.

**Table 1:** Transposable element insertions in ca. 5.7 Mb of orthologous genomic sequences from the coelacanth species *Latimeria chalumnae* and *L. menadoensis*.

TE classification		TE family	Common insertions	Species-specific insertions	
				*L. chalumnae*	*L. menadoensis*
Class I (retrotransposons)	LINE	CR1	286	6	9
		L1	11	-	2
		L2	4	1	-
	SINE	CoeG-SINEs	205	1	1
		Others	646	1	0
	LTR	Gypsy	24	1	-
		ERV*	0	- (solo LTR)	1 (element framed by 2 LTRs)
Class II (DNA transposons)		MITE-like	8	2	-
Composite insertions		CR1/SINEs	-	1	-
		CoeG-SINE/LF-SINE	-	-	1**
Other Class I and Class II families			1,879	-	-
Total			3,063	13	14

TE  =  Transposable Element; LINE  =  Long Interspersed Nuclear Element; SINE  =  Short Interspersed Nuclear Element; LTR  =  Long Terminal Repeat; CR1  =  Chicken Repeat 1; L1  =  LINE 1; L2  =  LINE 2; ERV  =  Endogenous Retrovirus; MITE  =  Miniature Inverted-repeat Transposable Element.

*The ERV insertion observed in *L. menadoensis* does not strictly correspond to an insertion polymorphism, the solo LTR observed at the orthologous site in *L. chalumnae* probably being the result of a recombination between the two LTRs framing the element (see main text).

**A composite insertion is observed in *L. menadoensis*, constituted by a Coeg-SINE flanked by two LF-SINEs in direct orientation. Only a “solo” LF-SINE is observed in *L. chalumnae*, suggesting deletion through homologous recombination between both LF-SINEs.

These “insertions” mostly comprise insertions *sensu stricto* but also a few deletions that occurred at the orthologous site in the other species.

**Table 2:** Structural features of species-specific transposable element insertions in ca. 5.7 Mb of orthologous genomic sequences from the coelacanth species *Latimeria chalumnae* and *L. menadoensis*.

Type of TE	Species with insertion	Insertion identifier	Insertion length (nt)	Target Site Duplication?	ORF(s)/Domain(s)/Specific features	Copy number in genomic sequences *	Element representation in the transcriptome **	Genomic position relative to next gene	Distance to the closest exon (kb) and corresponding gene
CR1	L. ch.	1	1622	AT	ORF2: RT	5 (2 with id ≥98%)	17	IGR	5.9 (*HOXD12*)
		2	1060	AT-rich region	ORF2: RT (partial)	1	0	Intron (exon 4–exon 5)	0.7 (exon 5) (*GRID1*)
		3	1097	GAGTCTTGTT	ORF2: RT (partial)	1	4	IGR	4.0 (*PCDHGC*)
		4	227	CTA	-	49 (5 with id ≥98%)	1	IGR	9.7 (*ighv14-1 (21)*)
		5	320	TTTAG	-	37 (5 with id ≥98%)	0	IGR	9.4 (vomeronasal 2 receptor)
		6	303	TATTAGG	-	1	0	IGR	>70.9 (*CALCOCO1*)
	L. me.	7	2845	ACTCA	ORF2: RT, APE (partial)	23 (4 with id ≥98%)	24	IGR	>9.0
		8	2821	AAT	ORF2: RT, APE (partial)	24	31	IGR	3.2 (*PCDHGC5*)
		9	1174	AAGTA	ORF2: RT (partial)	4	8	IGR	3.6 (*PCDHGC5*)
		10	1038	CCAT	ORF2: RT (partial)	74 (18 with id ≥98%)	10	IGR	18.3 (protocadherin gamma)
		11	862	GATTAA	ORF2: RT (partial)	86 (19 with id ≥98%)	6	Intron (exon 2–exon 3)	0.2 (exon 3) (*SRA1*)
		12	1398	TCTA	ORF2: RT (partial)	57 (15 with id ≥98%)	13	IGR	37.5 (*HOXB13*)
		13	1019	Poly-A region	ORF2: RT (partial)	1	0	Intron (exon 2–exon 3)	1.3 (exon 3) (*PCDHGC*)
		14	385	ND	-	110 (14 with id ≥98%)	0	Intron (exon 4–exon 5)	0.4 (exon 4) (*ighm*)
		15	387	CTATTCC	-	109 (12 with id ≥98%)	3	Intron (exon 2–exon 3)	6.2 (exon 2) (FAT tumor suppressor homolog)
L1	L. me.	16	2168	ACTAATCTTATTTTAA	Endonuclease (PFAM PF02994, “Transposase_22”) (partial)	2 (2 with id ≥98%)	20	IGR	41.6 (*hoxc1a*)
		17	1999	ND	RT	4	19	IGR	0.8 (*ighv14-1 (25)*)
L2	L. ch.	18	2219	G	RT	2	0	IGR	3.4 (*PCDHGC*)
CoeG-SINE	L. ch.	19	1362	ND	-	1	16	Intron (exon 4–exon 5)	0.6 (exon 5) (von Willebrand factor A domain containing 5A)
	L. me.	20	1018	ATTTT	-	1	0	IGR	18.0 (*EVX2*)
LF-SINE	L. ch.	21 (inserted within element 22)	391	TG	-	48	0	IGR	33.1 (uncharacterized protein)
Gypsy	L. ch.	22	896	CCCGCAGCGCCCCCCCCAGAGAAT	RT, no LTR	1	1	IGR	33.1 (uncharacterized protein)
ERV	L. me.	23	5091	AGAT	Gag, Pol, Env (partial), LTR	1	41	IGR	10.6 (*ighv14-1 (21)*)
MITE-like	L. ch.	24	225	CCT	-	2	0	IGR	6.4 (von Willebrand factor A domain containing 5A)
		25	1311	ATTTCAAG	Derived from a hAT transposon	1	5	IGR	2.8 (*CHRNB4)*
Composite insertion	L. ch.	26	2303	T	CR1 (RT, TSD “AGT”)/SINE (TSD “AAGT”)/LF-SINE/CoeSINE	1	5	IGR	90.7 (*CRHR2*)
	L. me.	27	1249	ND	CoeG-SINE/LF-SINE	1	0	IGR	>12.8

ORF  =  Open Reading Frame; L. ch.  =  *Latimeria chalumnae*; L. me.  =  *Latimeria menadoensis*; IGR  =  Intergenic Region; RT  =  Reverse Transcriptase; APE  =  Apurinic/Apyrimidic Endonuclease; LTR  =  Long Terminal Repeat; ND  =  Not Detected; TSD  =  Target Site Duplication; *Number of BlastN hits in the analyzed regions, with hit length ≥80% of insertion length and identity ≥80%, criteria that are classically used to define TE families; ** Number of BlastN hits against *L. menadoensis* testis transcriptome with hit length ≥80 nt and identity ≥95%.

**Figure 1:**
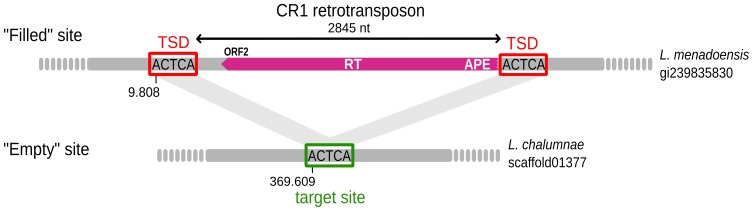
Example of a polymorphic insertion of a CR1 retrotransposon (element 7 in Table 2) present in *Latimeria menadoensis* but absent from *L. chalumnae*. Target Site Duplications (TSDs) are framed in red. CR1  =  Chicken Repeat 1; ORF  =  Open Reading Frame; RT  =  Reverse Transcriptase; APE  =  Apurinic/Apyrimidic Endonuclease.

Manual inspection of candidates for polymorphic insertions allowed us to exclude a number of false positives that corresponded to stretches of “N” in the *L. chalumnae* draft genome. Generally the length of such N stretches matched almost exactly the length of the insertion at orthologous positions in *L. menadoensis*, suggesting that they have been produced during the assembly phase. Pairwise alignment of “empty” and “filled” sites allowed us to define insertion boundaries, as shown in Figure 1. Several insertions presented evidence of degeneration (short size, truncated open reading frames, etc.) (Table 2).

Comparison between both coelacanth species led to the identification of 27 species-specific TE insertions, 13 in *L. chalumnae* and 14 in *L. menadoensis* (Tables 1 and 2). Insertion length ranged from 225 to 5,091 nt, with a mean of 1,363 nt. Insertions were classified according to TE ontology [16] using different specific characteristics of the superfamily, in particular the similarity with known TE-encoded proteins and the presence of specific structural features such as LTRs and TIRs. On the whole, identified polymorphic insertions mainly corresponded to CR1 non-LTR retrotransposons (6/13 and 9/14 insertions in *L. chalumnae* and *L. menadoensis*, respectively). A reverse transcriptase-encoding region, belonging to CR1 *ORF2* [38], was present in 3 and 7 of them, respectively, with only 3 cases (one in *L. chalumnae* and 2 in *L. menadoensis*) where it was apparently complete. The two longest *L. menadoensis* CR1 insertions with complete RT sequences showed 95% of nt identity (insertions 7 and 8; Table 2). They further contained, upstream of the RT, an APE (Apurinic/Apyrimidic Endonuclease) domain also belonging to *ORF2* (Figure 1). CR1 insertions appeared truncated in their 5' part, since they did not show any significant similarity with known *ORF1* sequences [38], [39]. While *ORF2* of insertion 8 presented several frameshifts and two stop codons, insertion 7 *ORF2* did not show any degeneration of the sequence. With the exception of a N-terminal deletion removing 140 amino acids of the APE domain due to 5'-truncation, the protein product predicted for this copy showed all amino acids thought to be important for its activity [39]. This suggests that this element might have been recently transposition-competent. Several short CR1 insertions corresponded to the extreme 3' part of the CR1 with no coding potential (insertions 4, 5, 6, 14 and 15, Table 2). Polymorphic insertions of other non-LTR retrotransposons from the L1 (two in *L. menadoensis*) and L2 (one in *L. chalumnae*) superfamilies were additionally detected, as well as SINEs (three in *L. chalumnae* and three in *L. menadoensis*), including the recently described CoeG-SINE family [27]. LTR retroelements were represented by a strongly corrupted copy of a Gypsy-like retrotransposon in *L. chalumnae* and an endogenous retrovirus in *L. menadoensis* (see below). For class II elements, only two insertions with a palindromic structure reminiscent of that of Miniature Inverted TEs (MITEs) were identified, one of them possibly derived from a hAT DNA transposon (as predicted using Censor). Finally, insertions 26 and 27 presented a composite structure. Insertion 26 comprised, sequentially, (i) a partial CR1 non-LTR retrotransposon with RT domain, framed by an “AGT” TSD, (ii) a possibly novel SINE element flanked by an “AAGT” TSD, (iii) half of a LF-SINE and (iv) a tRNA-derived SINE. Insertion 27 observed in *L. menadoensis* (Table 2) is formed by a CoeG-SINE flanked by two LF-SINEs in direct orientation, while a “solo” LF-SINE is found at the orthologous locus in *L. chalumnae*. This configuration suggests a deletion in *L. chalumnae* that occurred through a recombination between the two LF-SINEs framing the CoeG-SINE element in *L. menadoensis*.

TSDs, which are hallmarks of insertions consisting in few duplicated target site nucleotides (Figure 1), could be clearly identified in 20 out of 27 insertions. Two other insertions occurred in poly-A or AT-rich regions. Thirteen out of 16 CR1 insertions were flanked by TSDs, with no obvious sequence specificity for integration.

Altogether, polymorphic TE insertions covered a total of 13.3 kb in *L. chalumnae* and 23.5 kb in *L. menadoensis*, corresponding to approximately 0.23% and 0.41% of the genomic regions analyzed in the two coelacanth species. Hence, transposon activity is responsible for ca. 0.64% of genome divergence between both *Latimeria* species in the regions considered.

Among the 27 polymorphic sequences identified, six were found to be intronic, the others being located in intergenic regions (Table 2). While half of insertions were located more than 6 kb away from gene exons, five were closer than 1 kb from the next exon; in *L. menadoensis*, a CR1 element was inserted in an intron of the *SRA1* gene, about 200 base pairs next to the closest exon. Hence, some TE insertions are closely linked to coelacanth genes. Additional experiments will be required to determine if these insertions influence the function and evolution of neighbor genes.

### Structural and evolutionary analysis of new coelacanth endogenous retroviruses

**Figure 2:**
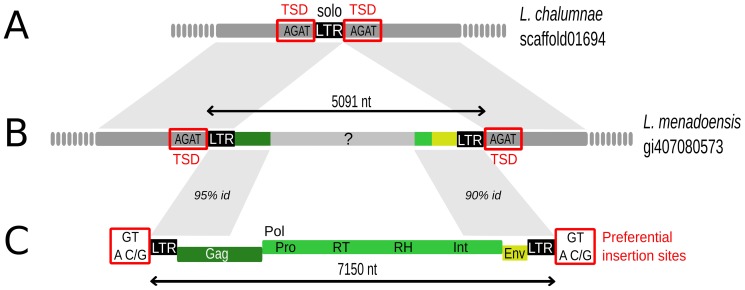
Structure of coelacanth endogenous retrovirus CoeERV1-1. (A) Solo-LTR observed in *L. chalumnae*. (B) Schematic representation of ERV insertion 23 found at the orthologous position in *L. menadoensis*. (C) Reconstructed structure of CoeERV1-1 in the *L. chalumnae* genome. TSD  =  Target Site Duplication; LTR  =  Long Terminal Repeats; Gag: ORF encoding protein for the viral capsid; Pol: ORF encoding proteins responsible for synthesis of the viral DNA and integration into host DNA, including protease (Pro), reverse transcriptase (RT), ribonuclease H (RH) and integrase (Int); Env: ORF encoding envelope protein.

The ERV insertion in *L. menadoensis* (insertion 23) corresponded to the largest of all identified polymorphic insertions (5,091 nt). This sequence showed a significant BlastX similarity (alignment of 113 amino acids with E-value  = 2.2e-10) with an integrase protein encoded by elements from the ERV1 family, which belongs to Epsilon retroviruses [40]. The ERV copy was delimited by two almost identical LTRs (462 nt, 99% of identity) and flanked by TSDs (“AGAT”) (Figure 2B). The orthologous region in *L. chalumnae* corresponded to a sequence of 462 nt almost identical to the LTRs of the ERV in *L. menadoensis* and flanked by the AGAT TSD sequences (Figure 2A). Hence, the *L. chalumnae* sequence corresponds to a so-called solo-LTR, formed through homologous recombination between the LTRs of the original retrovirus element, this eliminating one copy of the LTR as well as the intervening retrovirus sequence.

In order to better characterize the new coelacanth endogenous retrovirus, which was called CoeERV1-1, a consensus sequence was reconstructed from different copies found in the *L. chalumnae* genome (Figure 2C). This sequence contains LTRs (475 nt long) and a central region encoding Gag (viral capsid), Pol (polyprotein responsible for the synthesis of the viral DNA and its integration into host genome, including protease [Pro], reverse transcriptase [RT], ribonuclease H [RH] and integrase [Int]), and Env (envelope). Compared to this reconstructed sequence, the identified insertion (number 23 in Table 1) is strongly deleted, lacking major parts of the Gag and Pol domains (Figure 2 B–C). Most of the internal part of the insertion does not share any similarity with CoeERV1-1 and other known sequences. We detected 258 fragments of variable sizes similar to CoeERV1-1 in the whole *L. chalumnae* genome (identity >82%, E-value <10e-20), including four larger copies (ranging from 6,804 to 8,045 nt in size) with LTRs of variable sizes (from 444 to 551 nt) and all partial or complete ORFs. At least 10 of these 258 fragments corresponded to solo-LTRs, 8 being 475 nt long and presenting TSDs. Inspection of sixteen identified TSD sequences of CoeERV1-1 copies (ranging from 3 to 5 nt) suggested preference of insertion into target sites containing the “GT” (in 7 TSDs) or “AC/G” (in 10 TSDs) nucleotide motif (Figure 2C). Two TSDs contained both motifs while one did not contain any of them.

**Figure 3:**
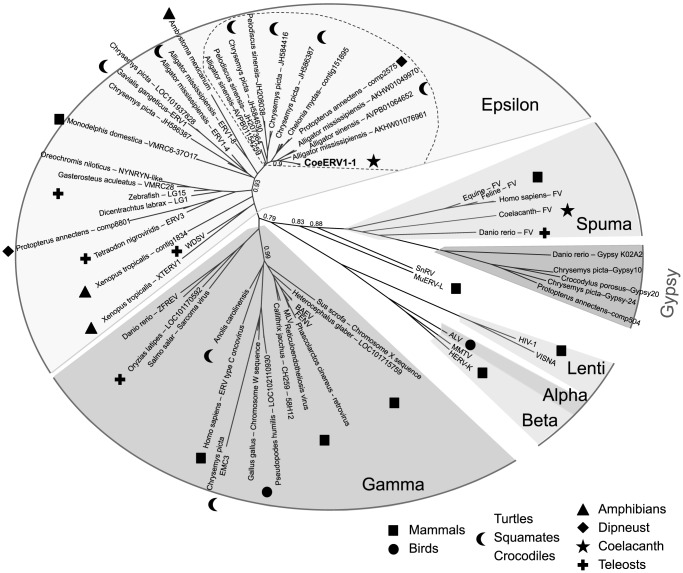
Phylogenetic relationship between coelacanth CoeERV1-1 and reptile retroviruses. Vertebrate retrovirus phylogeny was reconstructed on an alignment of RT (210 amino acids) using Maximum Likelihood with optimized parameters (best of NNI and SPR; optimized invariable sites [37]. Branch values represent supporting aLRT non-parametric statistics. The dashed line highlights the group of Epsilon viruses containing turtle, crocodile, coelacanth and lungfish sequences. Gypsy LTR retrotransposon sequences were used as an outgroup.

Retroviruses (RV) are classified in seven genera including Alpha-, Beta-, Gamma-, Delta-, Epsilon-, Lenti- and Spuma-viruses [40]. With six identified genera, the mammalian lineage presents the largest diversity of RV among vertebrates. To better understand the origin and evolution of the coelacanth ERV identified in this work, we performed phylogenetic reconstructions based on both RT (ca. 210 amino acids) and integrase core domain sequence alignments (ca. 132 amino acids) (Figure 3 and Figure S2). Coelacanth sequences were found to cluster in the Epsilon-virus group, one of the most spread branches of RV in vertebrates. Interestingly, CoeERV1-1 sequences were closely related to turtle and crocodile RV sequences, a result supported by both RT and integrase-based reconstructions (Figure 3 and Figure S2). Coelacanth ERV sequences share more than 3,000 nucleotides with over 64% of identity with alligator, crocodile and turtle sequences. This strong relatedness could suggest horizontal transfer (HT) between reptiles and coelacanths or infection of both lineages by a same subgroup of related retroviruses.

### Copy number and expression of TEs in coelacanth

In order to get more insight into the relative transposition activity of TEs identified as polymorphic in this work, we determined their copy number, including common insertions, by similarity search against the 5.7 Mb of orthologous sequences analyzed in both coelacanth species (filter: length ≥80% of the considered insertion length and sequence identity ≥80%; cf methods and Table 2). In 11 cases, including 4 CR1 non-LTR retrotransposons (insertions 2, 3, 6 and 13, Table 2), both CoeG-SINEs, both LTR elements, one MITE (insertion 25, Table 2) and the 2 composite insertions, we were only able to retrieve the query sequence with these filtering parameters, indicating the absence of other related copies of approximately the same size in the regions analyzed (Table 2). In contrast, other insertions were found reiterated, with copy numbers ranging from 2 to 110 (39 on the average). The highest hit numbers were obtained for CR1 sequences (insertion 14, up to 110 hits) and for the LF-SINE insertion (48 hits). Interestingly, eleven out of 15 CR1 species-specific insertions (insertions 1, 4–7, 9–12, 14 and 15) were very related one to each other: they showed a high degree of nt sequence identity (98–100%, Table S3) and grouped closely together in a CR1 molecular phylogeny (Figure S3). The CR1 subfamily formed by these sequences is responsible for 3.4% (105/3063) of insertions shared by both species, but constituted as much as 40% (11/27) of interspecific polymorphic insertions. Hence, a subfamily of CR1 retrotransposons has been very recently particularly active in coelacanth genomes, strongly contributing to genome divergence between both *Latimeria* species. L1 and L2 non-LTR retrotransposons as well as a MITE-like element (insertion 24) showed a more modest level of reiteration. The two L1 specific-insertions of *L. menadoensis* matched against two sets of sequences mutually non-overlapping, indicating that these two L1 correspond to distinct elements.

In order to determine if some of the polymorphic elements identified in this work (or elements closely related to them) might be expressed, and thus to get another clue on their putatively recent activity, TE insertions were used as queries against a *L. menadoensis* testis transcriptome (see methods). Table 2 presents for each insertion the number of Blast hits obtained with length ≥80 nt and identity ≥95%. Nine elements (2 in *L. chalumnae* and 7 in *L. menadoensis*) matched at least 10 times in the transcriptome: five CR1s including the most complete insertions 7 and 8, both L1s, one CoeG-SINE and the ERV, which presents the highest number of hits (41), probably because it is also the longest insertion, the hits being scattered all along its sequence. All nine elements are intergenic, apart from the CoeG-SINE, which is located in an intron. Common hits were obtained for the eleven CR1 elements of the subfamily previously described, with a total number of 29 distinct sequences in the transcriptome. This observation is congruent with the similarity search against genomic sequences suggesting stronger activity of this particular CR1 subfamily.

## Discussion

Comparative analysis of orthologous regions covering 5.7 Mb of the genome of the two extant coelacanth species strongly sustains the recent activity of TEs in this lineage, with the identification of 13 and 14 species-specific insertions in *L. chalumnae* and *L. menadoensis*, respectively. Insertions observed specifically in one or the other species suggest that these TE copies transposed after speciation, i.e. approximately within the last 6–8 million years. Alternatively, they might also correspond to insertion polymorphisms that predated the split between both species. Interestingly, with the exception of two MITE-like elements, most polymorphic insertions are retrotransposons. This indicates that DNA transposons are currently probably less active than retrotransposons in coelacanth genomes. CR1 LINEs represent most of recent insertions, with a more marginal contribution of tRNA-SINEs (CoeG- and LF-SINEs) and L1 and L2 LINEs. Hence, our results support relatively recent activity of CR1 retrotransposons and other LINE and SINE elements in coelacanth genomes. Retrotransposition of the non-coding tRNA-SINEs identified in this work might be catalyzed by autonomous CR1-LINEs or other LINEs. However, no significant similarity could be detected between the 3' part of LINE and SINE elements (data not shown). The more discrete presence of one ERV and one Gypsy element indicated that LTR retrotransposons also contribute to insertion polymorphism. Other types of repeats such as satellite sequences represent an additional form of genome divergence that was not considered in this study.

Target site duplications are hallmarks of insertions for most TEs. Many polymorphic insertions identified in this analysis showed recognizable TSDs, in particular many CR1 elements, suggesting that they transposed recently. Other arguments in favour of a recent/current transposition activity are the presence of very similar copies of a same element in the genome and the representation of TE sequences in transcriptomes, even if the latter does not necessarily imply functionality of the element. We have shown a particular enrichment in CR1 elements in coelacanth genomic sequences, as well as, to a lesser extent, the presence of SINEs and L1 and L2 retrotransposons. Similarity search against a testis transcriptome of *L. menadoensis* uncovered a high number of hits for CR1 elements as well as for L1 LINEs, CoeG-SINEs and an endogenous retrovirus. These results confirmed major activity of CR1 retrotransposons and the contribution of minor other types of TEs, mostly retroelements.

We also showed that homologous recombination between the LTRs of an endogenous retrovirus, or between adjacent tandem repeats, can contribute to genome divergence in coelacanths. This analysis led to the identification of CoeERV1-1, a so far unknown coelacanth Epsilon retrovirus, which is present in the genome of *Latimeria* species under the form of elements with two LTRs and partial or complete *gag*, *pol* and *env*, or as solo LTRs. Interestingly, phylogenetic analyses revealed a close relationship of coelacanth CoeERV1-1 with turtle and crocodile Epsilon retroviruses. This might be due to an horizontal transfer between coelacanths and reptiles, or to independent infections of both lineages by related retroviruses. The frequent high degree of nucleotide identity between both LTRs of a same copy, the high similarity between different copies and the presence of numerous copies with TSDs together suggest recent introduction into and/or recent transposition of CoeERV1-1 in the genome of coelacanths.

This study allowed to estimate the total number of species-specific insertions in the genomes of the two *Latimeria* species and to evaluate the impact of TEs on genome divergence in coelacanths. Our analysis, based on interspecific comparison of 5.7 Mb of orthologous genomic sequences (0.2% of the genome), indicated an average of 13.5 species-specific TE insertions. Hence, each *Latimeria* species might contain 6,500–7,000 TE insertions not found in the other species. This would mean that ca. 15,000 TE insertions are differentially present in both species, which diverged 6–8 mya. Strikingly, this value is similar to that reported for human and chimpanzee, which show 11,000 differentially present TE insertions (divergence 6 mya; [41], [42]). In term of DNA amount, TEs might be responsible for about 0.6% of genome divergence, i.e. for about 20 Mb of difference at the scale of the whole genome, between both coelacanth species. Importantly, this analysis particularly included gene-rich, euchromatic regions that were shown to contain slightly fewer TEs than the genome assembly average, which excludes highly repetitive regions. Hence, our estimation of TE contribution to coelacanth species-specific genome divergence might be an underestimation. In terms of population genetics, fixation of polymorphic TE insertions in both species might have been favored by the small effective population size in coelacanths [5]. Indeed, a small population size is thought to decrease the efficiency of purifying selection, allowing the fixation of neutral or even deleterious insertions by genetic drift [43]–[46].

To conclude, this work demonstrates that coelacanths possess active TEs that significantly contributed to post-speciation genome evolution. Hence the apparent morphological stasis of coelacanths might not be due to reduced TE activity, as previously proposed [21]. Our results also suggest that, beside transposition, other mechanisms such as ectopic homologous recombination and horizontal transfer might contribute to the plasticity of the coelacanth genome. This raises the question of the low impact of these mechanisms on the evolution of the coelacanth, or call again into question the postulated morphological stasis of *Latimeria*, which might not be supported by paleontological evidence [10].

## Supporting Information

Figure S1 **Protocol for insertion identification.** *L. chalumnae* (Lch) scaffolds and *L. menadoensis* (Lme) BACs are represented in blue and pink lines, respectively. Orthology relationships between Lme BACs and Lch genome (A) are determined by sequence comparison using MegaBlast [30] (B), as described in methods. TEs from orthologous fragments are then listed “face to face” (C) and further manually aligned to visualize orthologous insertions between the two species (D). Candidate species-specific insertions are further inspected by extracting and re-aligning corresponding “empty” and “filled” sites.
(TIF)

Figure S2 **Phylogenetic analysis of vertebrate retrovirus sequences.** Phylogeny is based on both reverse transcriptase (210 amino acids, left panel) and integrase core domain alignments (132 amino acids, right panel). Reconstruction was performed with the PhyML package [37] using Maximum Likelihood with optimized parameters (best of NNI and SPR; optimized invariable sites) and aLRT (SH-like branch supports).
(PDF)

Figure S3 **Phylogenetic analysis of CR1 species-specific and common insertions.** Phylogeny is based on an alignment of nucleotide sequences (1,387 sites) of 13 of the 15 CR1 species-specific insertions (SS, in red) and insertions common to both species (CI, in green). Two last specific insertions (SS3 and SS13) did not show enough significant similarity with other insertions to be unambiguously aligned. Reconstruction was performed with the PhyML package [37] using Maximum Likelihood with aLRT (SH-like branch support).
(PDF)

Table S1 **Coordinates of orthologous fragments in** ***L. menadoensis*** **BAC clones and in** ***L. chalumnae*** **genome.** Orthology links were determined by similarity search as described in methods. *L. chalumnae* genomic sequences were obtained from Ensembl (http://www.ensembl.org/; accession LatCha1 GCA_000225785.1), *L. menadoensis* BAC sequences from NCBI (http://www.ncbi.nlm.nih.gov/).
(PDF)

Table S2 **Coordinates and neighbouring genes of coelacanth species-specific insertions.** Insertions are numbered as in Table 2. Coordinates in bold correspond to insertions; coordinates in normal font correspond to orthologous empty sites.
(PDF)

Table S3 **Pairwise sequences comparison of the 15 specific CR1 insertions.** Comparisons were computed with the Blast bl2seq utility [34]. Each cell indicates, for the best hit obtained between the two compared sequences, the identity percentage, length and E-value of the match. Absence of significant matches are indicated by 'NAs'.
(XLS)
