# Supplementary figures and images for: Interspecies Insertion Polymorphism Analysis Reveals Recent Activity of Transposable Elements in Extant Coelacanths
Source: PLoS One. 2014 Dec 3;9(12):e114382. doi: 10.1371/journal.pone.0114382 (PMC4255032; doi:10.1371/journal.pone.0114382)

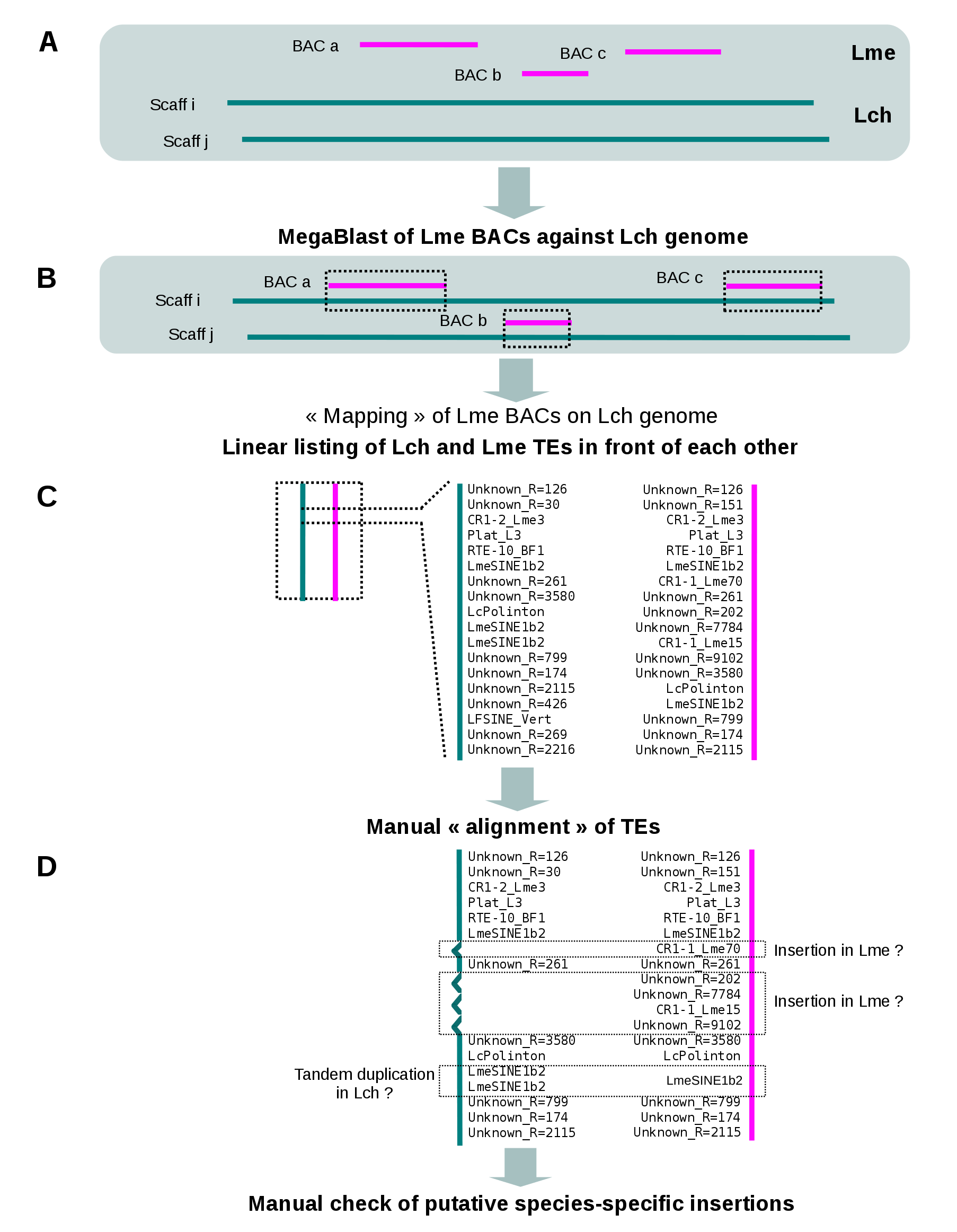

Supplement: Figure S1 — Protocol for insertion identification. L. chalumnae (Lch) scaffolds and L. menadoensis (Lme) BACs are represented in blue and pink lines, respectively. Orthology relationships between Lme BACs and Lch genome (A) are determined by sequence comparison using MegaBlast [30] (B), as described in methods. TEs from orthologous fragments are then listed “face to face” (C) and further manually aligned to visualize orthologous insertions between the two species (D). Candidate species-specific insertions are further inspected by extracting and re-aligning corresponding “empty” and “filled” sites. (TIF) [file pone.0114382.s001.tif]

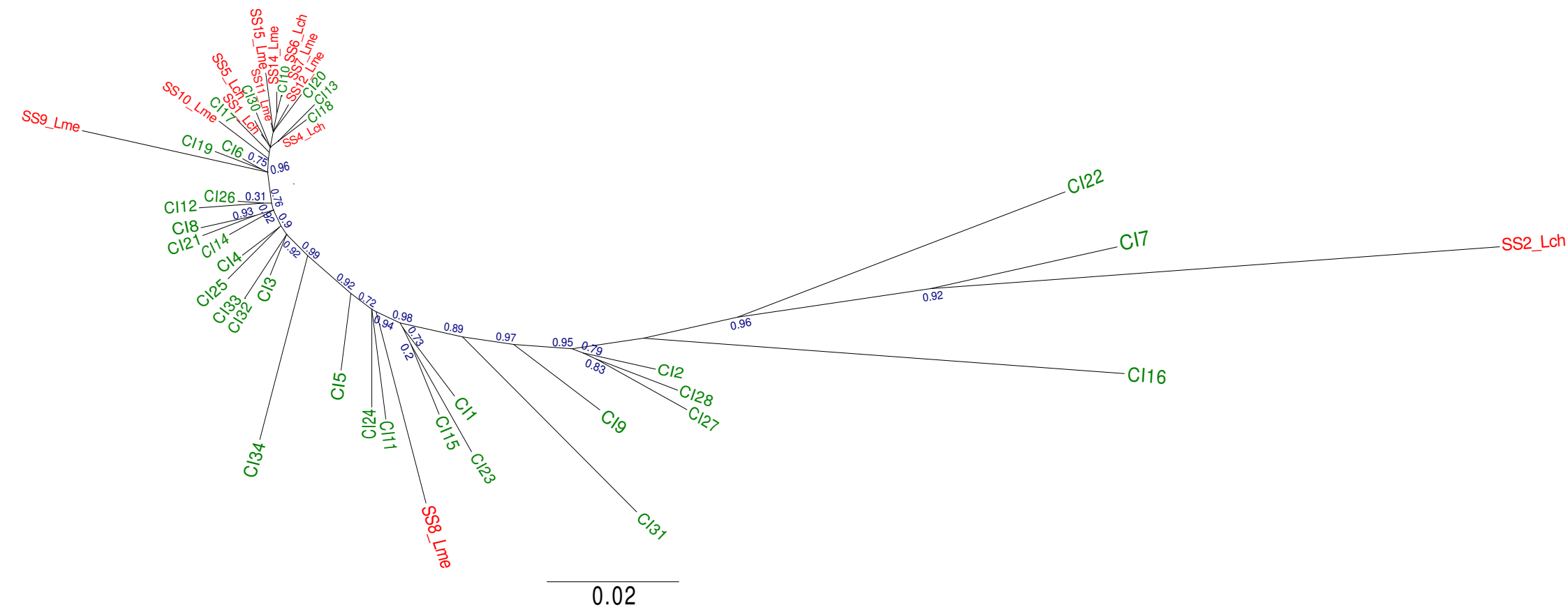

Supplement: Figure S2 — Phylogenetic analysis of vertebrate retrovirus sequences. Phylogeny is based on both reverse transcriptase (210 amino acids, left panel) and integrase core domain alignments (132 amino acids, right panel). Reconstruction was performed with the PhyML package [37] using Maximum Likelihood with optimized parameters (best of NNI and SPR; optimized invariable sites) and aLRT (SH-like branch supports). (PDF) [file pone.0114382.s002.pdf]

# Reverse transcriptase

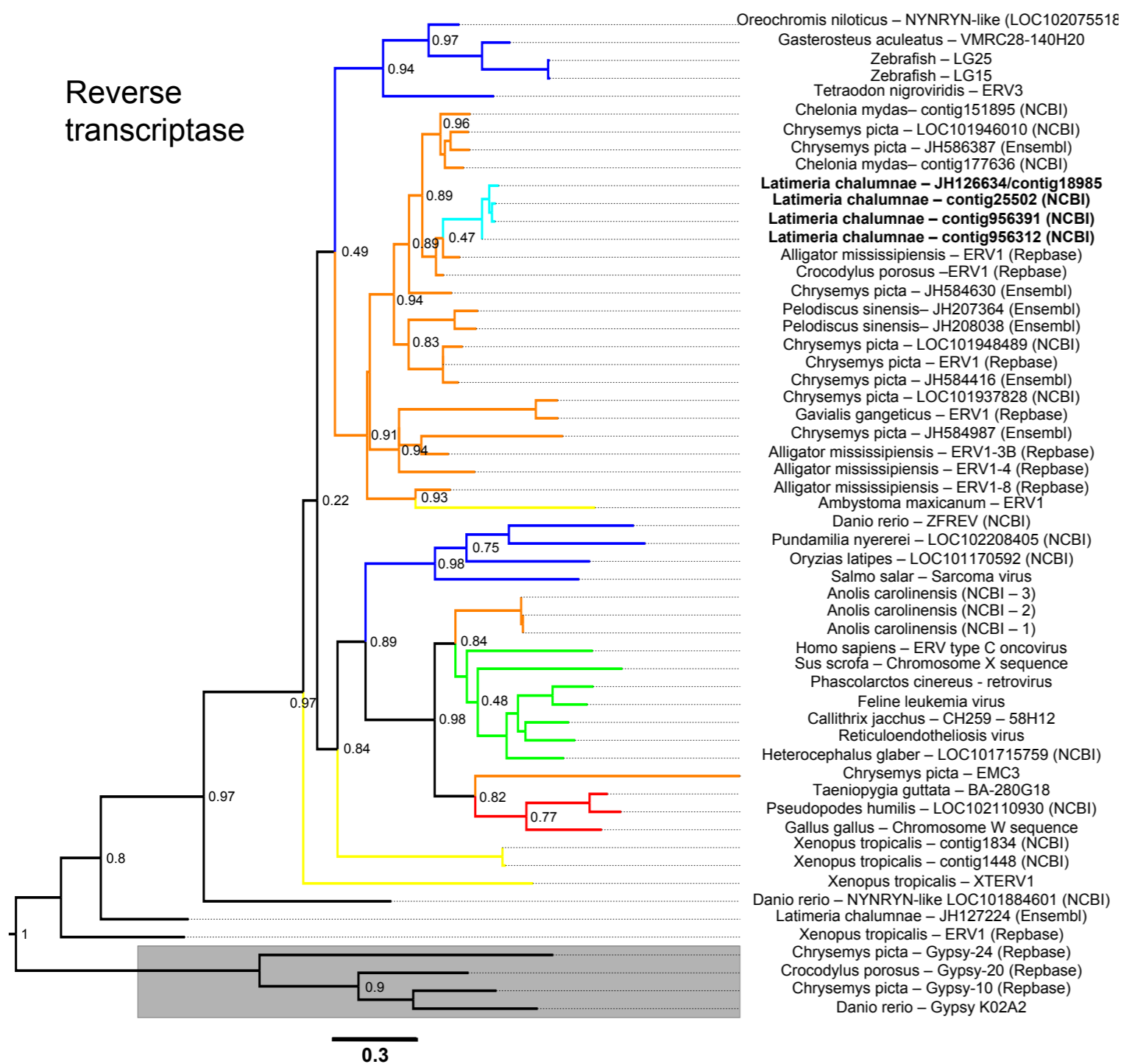

Integrase core domain

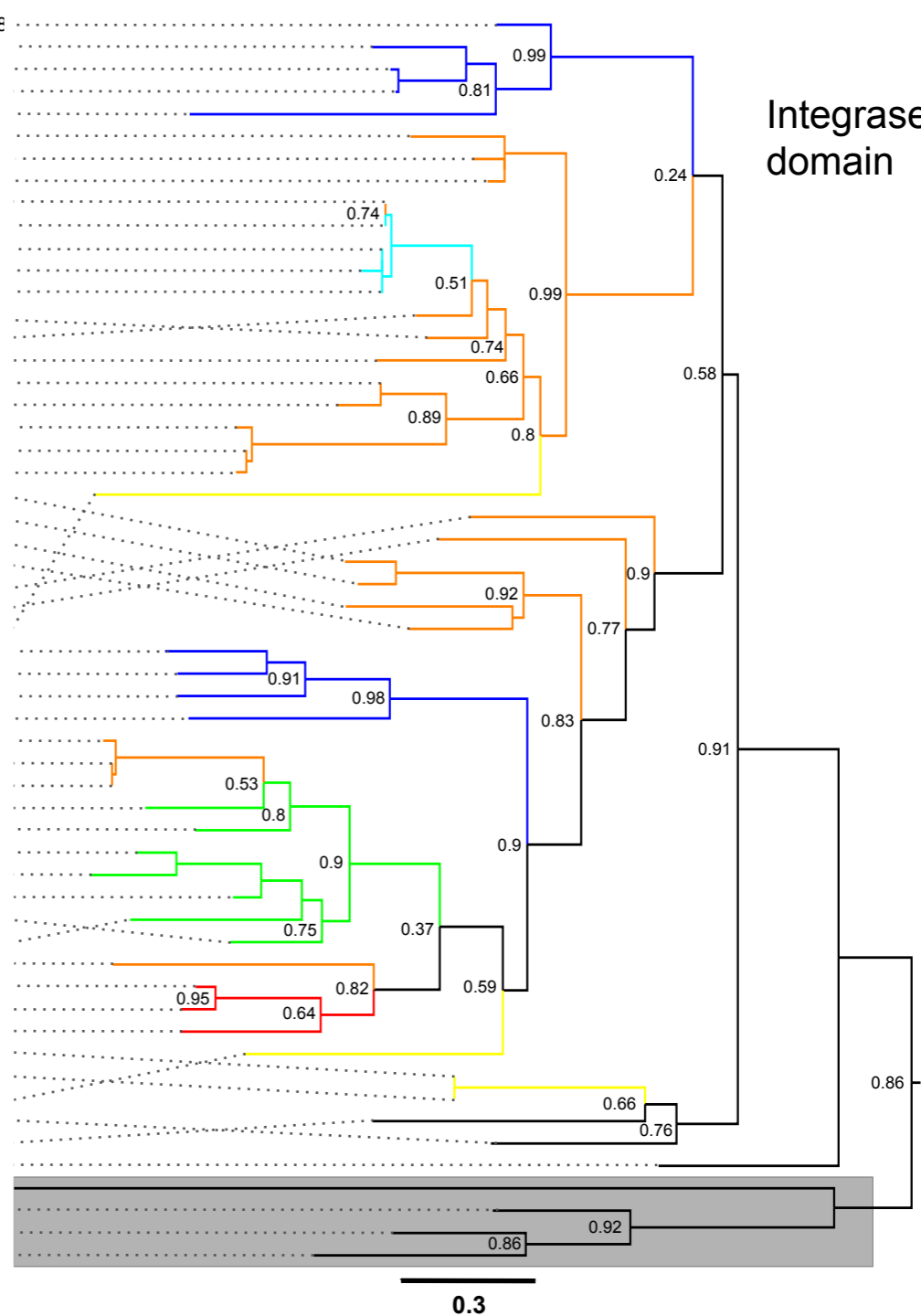

Supplement: Figure S3 — Phylogenetic analysis of CR1 species-specific and common insertions. Phylogeny is based on an alignment of nucleotide sequences (1,387 sites) of 13 of the 15 CR1 species-specific insertions (SS, in red) and insertions common to both species (CI, in green). Two last specific insertions (SS3 and SS13) did not show enough significant similarity with other insertions to be unambiguously aligned. Reconstruction was performed with the PhyML package [37] using Maximum Likelihood with aLRT (SH-like branch support). (PDF) [file pone.0114382.s003.pdf]
