# Supplementary material for: Interspecies Insertion Polymorphism Analysis Reveals Recent Activity of Transposable Elements in Extant Coelacanths
Source: PLoS One. 2014 Dec 3;9(12):e114382. doi: 10.1371/journal.pone.0114382 (PMC4255032; doi:10.1371/journal.pone.0114382)
Supplement: Table S1 — Coordinates of orthologous fragments in L. menadoensis BAC clones and in L. chalumnae genome. Orthology links were determined by similarity search as described in methods. L. chalumnae genomic sequences were obtained from Ensembl (http://www.ensembl.org/; accession LatCha1 GCA_000225785.1), L. menadoensis BAC sequences from NCBI (http://www.ncbi.nlm.nih.gov/). (PDF) [file pone.0114382.s004.pdf]

Latimeria chalumnae			*Latimeria menadoensis*			Match orientation (D: direct ; R: reverse)
Scaffold	Start	End	BAC	Start	End	
scaffold00009	3280929	3483227	GI:305644147	1	187889	R
scaffold00056	964812	1334312	GI:220898186	1081	373046	R
scaffold00059	1851179	2022554	GI:190886531	76	162566	R
scaffold00118	2047028	2214133	GI:239835829	1	166048	D
scaffold00119	237991	385773	GI:237406519	4976	147882	D
scaffold00130	383298	541448	GI:164698640	1460	160991	R
scaffold00150	1900790	2213195	GI:220898172	3028	310112	R
scaffold00155	44274	200545	GI:239835824	2666	156486	D
scaffold00254	1	176423	GI:50284580	11746	189301	D
scaffold00254	183290	281494	GI:50284579	64192	150004	D
scaffold00268	1477672	1759694	GI:220898198	120021	403307	R
scaffold00354	106073	392205	GI:407080572	1	277163	D
scaffold00384	1475305	1482115	GI:239835827	149505	156331	R
scaffold00402	700544	867644	GI:305644148	1	48164	R
scaffold00568	506160	684015	GI:239835822	1038	174368	D
scaffold00606	930938	1090454	GI:239835825	1	159151	D
scaffold00623	215249	743013	GI:220898210	2721	510561	R
scaffold00705	768998	874922	GI:239835826	29792	133856	D
scaffold00739	14474	181508	GI:193083250	3996	162395	R
scaffold00744	441927	611074	GI:239835828	1	168867	R
scaffold01111	1028	121776	GI:220898198	1876	110715	R
scaffold01303	366622	554618	GI:189459217	1354	181534	D
scaffold01377	359867	508183	GI:239835830	1	145595	D
scaffold01558	242036	417321	GI:50284581	1	170774	D
scaffold01558	440137	569572	GI:50253612	34992	161955	D
scaffold01681	112207	292021	GI:239835823	2188	173770	R
scaffold01694	223609	452608	GI:407080573	1	210648	R
scaffold01718	346125	497225	GI:296011776	3222	146768	D
scaffold01893	352535	452619	GI:170514516	8680	103197	D
scaffold01950	44085	185500	GI:239835827	1963	139437	R
scaffold01958	308291	432348	GI:239735715	3441	123477	D
scaffold03191	32534	87846	GI:66912372	29226	85540	R
scaffold04994	1	55609	GI:170514516	109303	160968	R
scaffold06915	81	12807	GI:66912372	13200	26009	D
scaffold12374	692	4651	GI:239835826	8731	12694	R
