# Supplementary material for: Interspecies Insertion Polymorphism Analysis Reveals Recent Activity of Transposable Elements in Extant Coelacanths
Source: PLoS One. 2014 Dec 3;9(12):e114382. doi: 10.1371/journal.pone.0114382 (PMC4255032; doi:10.1371/journal.pone.0114382)
Supplement: Table S2 — Coordinates and neighbouring genes of coelacanth species-specific insertions. Insertions are numbered as in Table 2. Coordinates in bold correspond to insertions; coordinates in normal font correspond to orthologous empty sites. (PDF) [file pone.0114382.s005.pdf]

Insertion ID	Coordinates of insertion/ empty site in *L. chalumnae*	Coordinates of insertion/ empty site in *L. menadoensis*	Closest gene (*L. chalumnae* ortholog identifier)
1	**Scaffold00623:444,442-446,063**	GI:220898210:285,592	ENSLACG00000009639 (*HOXD12*)
2	**Scaffold00118:2,061,845-2,062,904**	GI:239835829:14,875	ENSLACG00000017224 (*GRID1*)
3	**Scaffold00254:143,688-144,784**	GI:50284580:156,232	ENSLACG00000003645 (*PCDHGC*)
4	**Scaffold01694:371,635-371,861**	GI:407080573:70,685	ENSLACG00000008667 (*ighv14-1 (21)*)
5	**Scaffold01958:325,272-325,591**	GI:239735715:18,223	ENSLACG00000008317 (vomeronasal 2 receptor)
6	**Scaffold01111:49,937-50,239**	GI:220898198:68,977	ENSLACG00000008634 (*CALCOCO1*)
7	Scaffold01377:369,609	**GI:239835830:9,808-12,652**	No gene on scaffold01377
8	Scaffold00254:219,610	**GI:50284579:89,241-92,061**	ENSLACG00000006212 (*PCDHGC5*)
9	Scaffold00254:225,605	**GI:50284579:95,826-96,999**	ENSLACG00000007011 (*PCDHGC5*)
10	Scaffold00254:159,054	**GI:50284580:170,519-171,556**	ENSLACG00000003645 (*PCDHGC*)
11	Scaffold01558:304,507	**GI:50284581:55,931-56,792**	ENSLACG00000007697 (*SRA1*)
12	Scaffold00056:1,246,007	**GI:220898186:86,058-87,455**	ENSLACG00000015543 (*HOXB13*)
13	Scaffold00254:99,438	**GI:50284580:111,150-112,168**	ENSLACG00000003645 (*PCDHGC*)
14	Scaffold01694:291,311	**GI:407080573:145,750-146,134**	ENSLACG00000007502 (*ighm*)
15	Scaffold01558:396,181	**GI:50284581:149,127-149,513**	ENSLACG00000008820 (FAT tumor suppressor homolog)
16	Scaffold00268:1,507,311	**GI:220898198:371,470-373,637**	ENSLACG00000016368 (*hoxc1a*)
17	Scaffold01694:~445,237	**GI:407080573:~4,900-6,898**	ENSLACG00000009775 (*ighv14-1 (25)*)
18	**Scaffold00254:219,832-222,050**	GI:50284579:92,281	ENSLACG00000006212 (*PCDHGC*)
19	**Scaffold00354:~368,219-369,580**	GI:407080572:~254,678	ENSLACG00000008465 (von Willebrand factor A domain containing 5A)
20	Scaffold00623:265,988	**GI:220898210:249,373-250,390**	ENSLACG00000010437 (*EVX2*)
21	**Scaffold00402:858,209-858,599**	GI:305644148:8,349	ENSLACG00000013670 (uncharacterized protein)
22	**Scaffold00402:858,000-859,286**	GI:305644148:8,349	ENSLACG00000013670 (uncharacterized protein)
23	Scaffold01694:351,066 (solo LTR)	**GI:407080573:91,275-96,365**	ENSLACG00000008667 (*ighv14-1 (21)*)
24	**Scaffold00354:388,393-388,617**	GI:407080572:273577	ENSLACG00000008465 (von Willebrand factor A domain containing 5A)
25	**Scaffold00739:136,634-137,944**	GI:193083250:45,783	ENSLACG00000004838 (*CHRNB4)*
26	**Scaffold01681:185,790-188,092**	GI:239835823:103,074	ENSLACG00000001393 (*CRHR2*)
27	Scaffold03191:46,696	**GI:66912372:70,080-71,328**	No gene on scaffold03191
